# Aurora kinases: Generators of spatial control during mitosis

**DOI:** 10.3389/fcell.2023.1139367

**Published:** 2023-03-13

**Authors:** Aamir Ali, P. Todd Stukenberg

**Affiliations:** Department of Biochemistry and Molecular Genetics, School of Medicine, University of Virginia, Charlottesville, VA, United States

**Keywords:** Aurora kinases, chromosome biorientation, kinetochore-microtubule interactions, kinase gradients, mitosis, nuclear envelope reformation, mitotic spindle, micronuclei

## Abstract

Cell division events require regulatory systems to ensure that events happen in a distinct order. The classic view of temporal control of the cell cycle posits that cells order events by linking them to changes in Cyclin Dependent Kinase (CDK) activities. However, a new paradigm is emerging from studies of anaphase where chromatids separate at the central metaphase plate and then move to opposite poles of the cell. These studies suggest that distinct events are ordered depending upon the location of each chromosome along its journey from the central metaphase plate to the elongated spindle poles. This system is dependent upon a gradient of Aurora B kinase activity that emerges during anaphase and acts as a spatial beacon to control numerous anaphase/telophase events and cytokinesis. Recent studies also suggest that Aurora A kinase activity specifies proximity of chromosomes or proteins to spindle poles during prometaphase. Together these studies argue that a key role for Aurora kinases is to provide spatial information that controls events depending upon the location of chromosomes or proteins along the mitotic spindle.

## Introduction

Gradients are used in biology to provide spatial information, for example, morphogen gradients provide information to specify the location of cells in developing tissues to define cell fates that pattern embryos. Aurora B kinase generate a gradient of kinase activity in the central region of anaphase spindles (Fuller et al., 2008), which suggested the exciting idea that Aurora kinases generate intracellular spatial information for anaphase events. In this review we will discuss recent findings that demonstrate how cells use this gradient of Aurora kinase activity during anaphase to coordinate the release of kinetochores and reformation of the nuclear envelopes with the movement of chromosomes towards the poles. We will also highlight research findings that show how during prometaphase Aurora A provides spatial information about proximity to spindle poles to control the release of both improper kinetochore-microtubule attachments and dynein cargoes. Together these studies demonstrate that the role of Aurora kinases is to provide spatial information that imputes the location of molecules along the mitotic spindle to coordinate cell division events.

Spatial regulation in interphase is dominated by the containment of reactions to membrane bound and membrane-less organelles. During mitosis these organelles are largely broken down and thus new mechanisms for spatial control are required (Rai et al., 2018). At the center of mitosis is the mitotic spindle, which occupies most of a somatic cell volume and it’s large size is required for its function of separating chromosomes to two daughter cells. We will discuss mechanisms that cells have evolved to measure the location of components along the spindle and highlight how cells use the spatial information to control the mitotic events.

During anaphase the spindle elongates dramatically and a group of microtubule bundles emerge between the segregating chromosomes, collectively known as the spindle midzone (Wadsworth, 2021). The midzone contains microtubule-bundling and capping proteins, kinesin motors and key proteins required for cytokinesis. A component of the midzone is the Chromosome Passenger Complex (CPC), comprising of Aurora B as the kinase catalytic subunit. There is a second pool of the CPC on the microtubule bundles at the plasma membrane at the presumptive location that defines the cytokinesis furrow (Landino and Ohi, 2016; Landino et al., 2017). The CPC is required to complete cytokinesis but distinguishing the roles of the midzone or cortical pools in this particular process is still an area of intense research since there are no clean ways to separate the two pools of the CPC and there is much redundancy in cytokinetic pathways.

**FIGURE 1:**
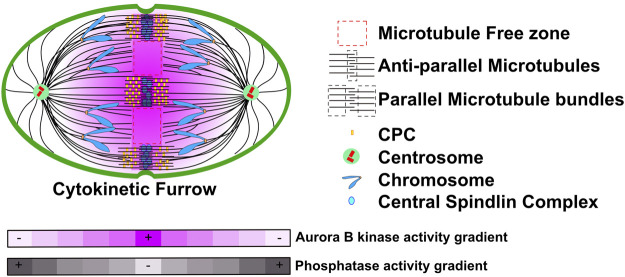
A model representation of CPC localization and Aurora B activity during anaphase. During anaphase the CPC localizes to two bundles of microtubules in the region between the segregating chromatids. First, spindle midzones are comprised of two parallel bundles of microtubules that have antiparallel crosslinks in the center. The CPC also binds a second set of microtubule bundles in the vicinity of cell cortex where the cytokinetic furrow will form. However, there must also be soluble pools of the CPC that are not easily visualized as Aurora B activity forms gradients that emanate from these pools. These gradients are highest in the central region of the spindle and decrease toward both poles. Grey heat map represents a potential Phosphatase activity gradient that we speculate could emanate from poles. Figure representation: Cell cortex, green; Spindle poles, green circle; Centrioles, red cylinders; Chromosome arms, blue; Kinetochores, Orange rectangles; Microtubules, black lines; Central Spindlin complex, blue ovals; CPC, yellow rectangles; Parallel and Antiparallel microtubule crosslinks are highlighted within dash black lines; Microtubule free zones are highlighted within dash red lines; Pink heat map represents Aurora B kinase activity gradient. Abbreviation: CPC, Chromosomal Passenger Complex.

The CPC is highly localized to midzone bundles of microtubules as well as to the bundles of microtubules that lie at the plasma membrane where the cytokinetic furrow is expected to form (Figure 1). The elegant experiments from *Xenopus* and *Zebrafish* embryo’s suggested that the CPC at spindle midzone precedes and specifies the microtubule bundles at the plasma membrane (Wuhr et al., 2009; Wuhr et al., 2010; Field et al., 2011; Mitchison et al., 2012). Surprisingly, the kinase activity of Aurora B is not constrained to these bundles of microtubules, rather it can be measured on the chromosomes that move along the microtubules towards the poles (Fuller et al., 2008), even though the kinase levels on chromosomes are too low to be measured by immunofluorescence. Importantly, kinase activity is highest on the chromosomes that are in center of the spindle and then decreases as a function of distance towards both spindle poles. Thus, there is a gradient of Aurora B kinase activity that is highest in the center of the anaphase spindle (Figure 1). The activity has been measured using Aurora B sensors targeted to microtubules, chromosomes and centromeres and on substrates on chromosomes and kinetochores and thus is quite robust (Fuller et al., 2008; Tan and Kapoor, 2011; Ye et al., 2016). It was established that Aurora kinase must be active during anaphase to generate the gradients, but it is not known if there is an opposing gradient of phosphatase activity, which could also contribute to the measured gradients of activity on kinase substrates (Figure 1). We will refer to the gradients as Aurora B kinase gradients but it is important to appreciate that the gradients may be formed both from Aurora kinases and opposing phosphatases to generate phosphorylation potential that decreases as a function of distance from the center of the cell.

**FIGURE 2:**
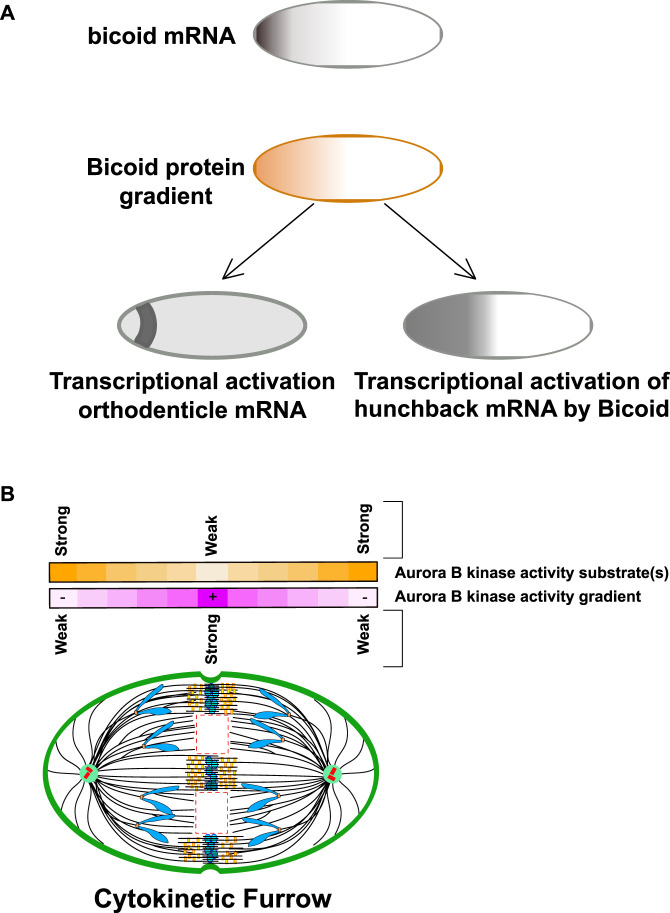
Intracellular gradients can transmit spatial information through relative binding affinities. **(A)** Bicoid protein gradients provide spatial information for anterior-posterior patterning of *drosophila* early embryonic syncitia. Bicoid mRNA is localized to the anterior pole of the *Drosophila* oocyte. Upon fertilization, the mRNA is translated generating that is high in the anterior pole. Bicoid is a transcription factor that binds a consensus sequence in regulatory elements of patterning genes. One such gene is orthodenticle which contains a poor binding site for bicoid and is only translated in the most anterior regions. In contrast hunchback has a better Bicoid binding site and has a broader transcriptional region in the anterior pole of the embryo. Note hunchback is also transcribed in the posterior pole in a bicoid independent manner but this is not shown for clarity. Figure adapted from Porcher and Dostatni (2010). **(B)** Kinase-phosphatase activity gradients could generate spatial information by phosphorylating substrates in different regions of the gradient Purple line: CPC component Aurora B kinase generates a kinase gradient centered at the spindle midzone. The kinase activity is highest at the spindle midzone and decreases with the distance towards the spindle pole. Yellow line: Analogous to Bicoid protein activity, substrates with weak kinase consensus site may only be phosphorylated in the central region where the activity is highest while better kinase substrates would be phosphorylated in a broader region of the gradient. Figure representation: Cell cortex, green; Spindle poles, green circle; Centrioles, red cylinders; Chromosome arms, blue; Kinetochores, orange rectangles; Microtubules, black lines; Central Spindlin complex, blue ovals; CPC, yellow rectangles; Microtubule free zones are highlighted within dash red lines. Abbreviation: CPC, Chromosomal Passenger Complex.

It is worth speculating about how gradients of kinase activity might provide spatial information for cells. An interesting example of a well-studied intracellular gradient is the early embryonic *drosophila* syncytium, where gradients of proteins and resulting activities can be easily measured since they emerge in a large single cell that has many nuclei in a single cytoplasm (Figure 2A). Bicoid is a transcription factor important for anterior-posterior patterning. Bicoid binds a consensus site in enhancers that define the anterior segments of the embryo (Porcher and Dostatni, 2010; Lucas et al., 2018). The RNA for bicoid is highly localized to the anterior pole, which forms a gradient of the bicoid protein that is high at the anterior pole and decrease towards the posterior pole. The bicoid gradient provides spatial information because the cis DNA sequences that bicoid protein binds to in the enhancers have different affinities for bicoid protein. Specifically, enhancers that have poor bicoid consensus binding motifs (such as the gene for orthodenticle) are only activated in nuclei that have high bicoid protein and thus are close to the anterior pole. In contrast, enhancers containing tight bicoid binding sites (such as hunchback) are also bound by bicoid in nuclei that are much farther from the anterior pole and thus specify genes for more posterior segments. We suggest that kinase gradients could be used to regulate substrates using this paradigm. Specifically, Aurora B substrates that are poorly phosphorylated would have a central phosphorylation pattern on the anaphase spindle, while those that have a good kinase consensus phosphorylation motifs would be phosphorylated in a broader region of the gradient (Figure 2B). In this review we will highlight new studies that show that Aurora gradients control events at distinct regions of the spindle, however, an important area of investigation will be to test this model by, for example, generating subtle modulations of the consensus sites of the proteins we will discuss below to determine if this can move events to different spindle locations.

## Cellular activities regulated by the anaphase gradients of Aurora B kinase activity

The original paper that described the anaphase gradient of Aurora B kinase activity demonstrated that perturbations that change the location of the cytokinetic furrow in turn changed the shape of the gradient, which suggested that the gradient has a role in cytokinesis (Fuller et al., 2008). They also targeted FRET sensors to kinetochores to show that Aurora B activity on the kinetochores of segregating chromosomes decreased as chromosomes moved towards the poles (Fuller et al., 2008). However, the function of this gradient has remained mysterious for over 15 years. A recent series of findings are solidifying the concept that the gradients provide spatial information that is used 1) to order the events of anaphase, 2) provide robustness to chromosome segregation and 3) may even regulate processes that prevent tumor formation.

**FIGURE 3:**
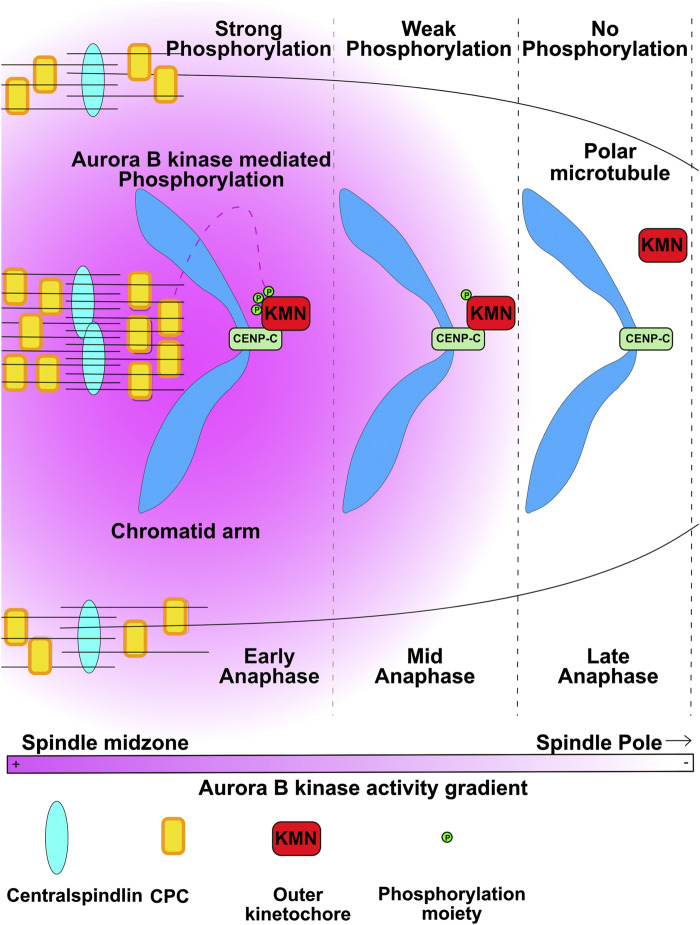
Kinetochores disassembly is controlled by chromosomes traversing from regions of high Aurora B activity to low activity. Aurora B kinase phosphorylates the DSN1 subunit of KMN (KMN) to allow KMN to bind the CENP-C protein to assemble kinetochores. As segregating chromatids are pulled to areas of low Aurora activity the KMN proteins are dephosphorylated and kinetochores disassemble. Figure representation: Chromosome arms, Blue; CENPC, light green rectangles; KMN, red rectangles; Phosphorylation moiety, green circles, Microtubules, black lines; Central Spindlin complex, Blue ovals; CPC, yellow rectangles; Pink heat map represents Aurora B kinase activity gradient. Abbreviation: CPC, Chromosomal Passenger Complex; KMN, KNL1-Mis12-Ndc80 outer kinetochore complex. Figure adapted from Papini et al. (2021).

In vertebrate cells the outer kinetochore proteins disassemble from centromeres after sister chromatids separate in late anaphase/early telophase. This disassembly is believed to be required to release kinetochore fiber microtubules to allow proper formation of the nuclear envelope. Kinetochore associated microtubules are bound by the Ndc80 subassembly of the KMN complex and the assembly of KMN onto a kinetochore requires Aurora B phosphorylation (Yang et al., 2008; Dimitrova et al., 2016; Emanuele et al., 2008). Dephosphorylation of kinetochores by PP1 was shown to disassemble the Ndc80 complex from purified chromosomes suggesting that dephosphorylation is sufficient to release KMN (Emanuele et al., 2008). In a recent study, the two groups used a specific phosphoantibody against the phosphosite on the KMN complex that is required to assemble kinetochores in anaphase cells. Phosphorylation on this site decreased as a function of distance from the anaphase spindle midzone (Papini et al., 2021). Another Aurora B phosphorylation on the N-terminus of Knl1 also shows this loss of phosphorylation as a function of distance from the equator (Sen et al., 2021). These findings suggest that these site are dephosphorylated when chromosomes move out of the region of high Aurora activity as they move towards the spindle poles during anaphase (Figure 3). The loss of DSN-1 phosphorylation was correlated with the disassembly of kinetochore proteins which occurs short distances farther towards the poles than the dephosphorylation of KMN (Papini et al., 2021). Thus, these findings argue that the Aurora kinase gradients provide spatial information that controls when and where kinetochores are disassembled.

A distinction of a spatially-control over the global-control system such as the cell cycle is that the location of each chromatid can be independently assessed and chromosomes that remain in the center of the spindle can maintain their kinetochore-microtubule attachments much longer than those that rapidly segregate. In fact, chromosomes that have improper kinetochore-microtubule attachments and remain in the central area of the spindle retain their kinetochores longer than the chromosomes that have moved to poles, which allows many of these chromosomes to eventually move to poles to limit the number of missegregated chromosomes during mitosis (Orr et al., 2021; Papini et al., 2021). This “Chromosome Autonomous Regulation” is another theme of Aurora B kinase regulation that is involved in initiating a number of processes that are independently controlled on each chromosome during prometaphase including the spindle assembly checkpoint and controlling kinetochore-microtubule attachments (Trivedi and Stukenberg, 2016).

## Micronuclei, which are a hallmark of chromosomal instability (CIN) in cancer, may be prevented by the Aurora kinase gradient

**FIGURE 4:**
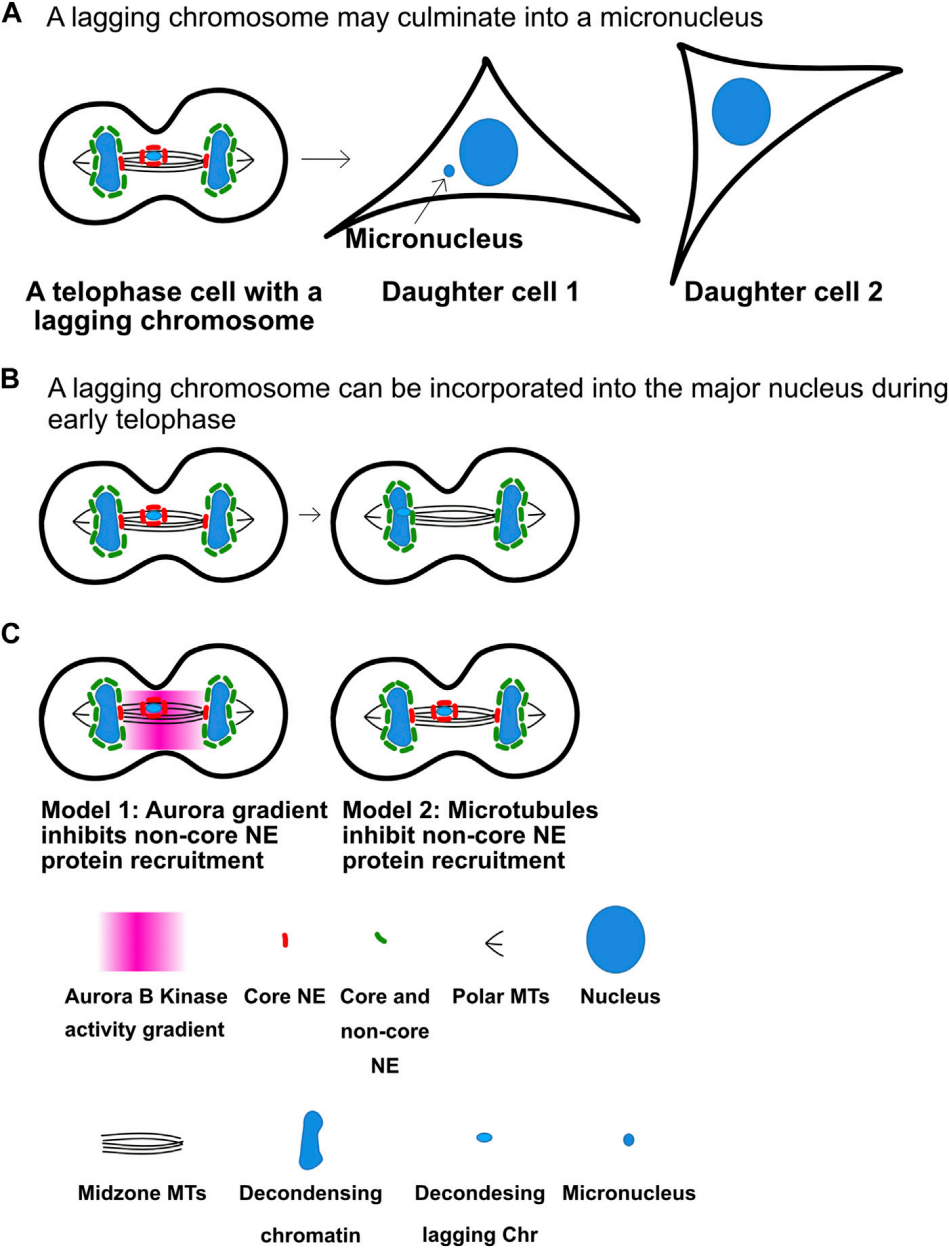
Anaphase Aurora B kinase gradients may regulate nuclear envelope assembly to limit micronuclei formation. **(A)** Decondensing chromosomes may culminate in the formation of micronuclei in the next G1. **(B)** Lagging chromosomes between chromosome masses do not recruit non-core nuclear envelope proteins, which allows them to be incorporated in the main nucleus. As cells enter telophase nuclear envelope formation begins on all chromatin masses. Segregated chromosome masses can recruit both core and non-core nuclear envelope proteins (Green) to form normal nuclear envelopes. However, lagging chromosomes only bind a set of nuclear envelope proteins known as core proteins (Red) but cannot recruit non-core proteins. This is believed to have two consequences. First, it allows chromosomes that arrive late to incorporate into the major nucleus and prevent micronuclei formation. Second, if the lagging chromatids are not incorporated in the major nucleus by telophase they become micronuclei that are deficient in the ability to form nuclear pores and also easily rupture. **(C)** There are two models for the mechanisms that prevent the non-pore nuclear envelope proteins from assembling. The first model posits that the Aurora B gradient prevents the binding. The second argues that bundles of microtubules prevent the non-pore proteins from binding. Note the in both model’s chromosomes that are not between segregating chromosomes cannot recruit non-pore proteins and become micronuclei. It will be important to determine the mechanisms to distinguish between the two models. Figure representation: De-condensing chromosomes, blue; Nucleus, large blue ovals; Micronucleus, small blue oval; Microtubules, black lines; Core NE proteins, red bean shape; Core and non-core NE proteins, green bean shape; Pink heat map represents Aurora B kinase activity gradient. Abbreviation: NE, Nuclear envelope.

Lagging anaphase chromosomes or chromatids can initiate many of the chromosome changes that drive cancer (Leibowitz et al., 2015; Liu et al., 2018; Liu and Pellman, 2020). A micronucleus is a smaller nucleus in the same cell physically separated from the normal nucleus. Micronuclei are a hallmark of tumors and the chromosomes inside them are genomically unstable and accumulate large numbers of double strand breaks. In fact, micronuclei are believed to underlie chromothripsis, extrachromosomal circular amplifications and bridge-fusion breakage cycles all of which are hallmarks of cancer (Leibowitz et al., 2015; Liu and Pellman, 2020). Lagging anaphase chromatids are precursors to micronuclei and they form when chromosomes or chromatids are not segregated to the poles when the nuclear envelopes reform (Figure 4A). At the end of anaphase, the separated chromatids at the two poles are in close proximity to each other and nuclear envelope forms around each group of chromatids to become separate nuclei (Afonso et al., 2014a; Afonso et al., 2014b). In contrast, if a chromatid’s movement is delayed during anaphase such that it reaches the pole after formation of the major nucleus then it must form its own nuclear envelope, generating a micronucleus. A series of findings have suggested that a key role of the Aurora B gradient is to prevent lagging chromatids from becoming micronuclei (Afonso et al., 2014a; Orr et al., 2021).

Maiato and others have shown that most lagging chromosomes do not culminate into micronuclei, which they argue suggests that cells have active mechanisms that prevent them (Orr et al., 2021). They also showed that the nuclear envelope reformation (NER) in telophase is not controlled by a clock since manipulations that slow chromosome movement velocities also delay NER. They have shown that Aurora gradients control a number of events to ensure lagging chromosomes have a chance to re-enter the major nucleus (Figure 4B). They provide complementary data to the Higgins group that Aurora kinase gradients preserve kinetochore-microtubule attachments allowing microtubule-dependent forces to continue to pull the chromatids towards the poles. In addition, they argue that the gradients control the formation of complete nuclear envelopes around chromatin. However, Pellman group has suggested an alternative model which suggests that it is the location of chromosomes or chromatids near microtubule bundles (such as midzones) that prevents mature nuclear envelope formation (Figure 4C). In support of this, microtubule stabilization was shown to prevent nuclear envelope formation in the presence of Aurora kinase inhibitors (Liu et al., 2018; Liu and Pellman, 2020). Subsequently the mAiato group demonstrated that NE can form on lagging chromosomes in midzones if they prevented the localization of the CPC to midzones, arguing again for a role for the gradient in preventing NER (Orr et al., 2021). Both groups argue that immature nuclear envelopes form around lagging chromosomes, which can explain why micronuclei have defective nuclear transport and nuclear envelopes that often rupture which can lead to the double stranded breaks that drive chromothripsis and other cancer related chromosome changes.

It is currently a matter of debate whether these mechanisms have evolved as a mechanism to allow lagging chromosomes to incorporate into the major nucleus (Liu and Pellman, 2020). An alternative model is that the incorporation of lagging chromosomes into the major nucleus is a matter of timing such that the lagging chromosomes that arrive before nuclear envelope reformation occurs around the major nucleus are incorporated while those that arrive later become micronuclei. It is also possible that cells have evolved these mechanisms to identify cells that have late lagging chromosomes and target them for cGAS/STING mediated cell death. If true this would represent a genomic stability mechanism that could be altered during cancer progression. The levels of the CPC and many of its regulators are overexpressed in highly aneuploid tumors (Pfister et al., 2018) and it will be important to determine if these pathways are affected in tumor cells and drive tumorigenesis.

The McAinsh group identified a separate mechanism by which Aurora B kinase can prevent micronuclei (Sen et al., 2021). They suggest that Aurora B corrects improper kinetochore-microtubule attachments in anaphase. They performed a series of elegant time-lapse experiments using lattice light sheet microscopy to follow lagging anaphase chromosomes. These experiments suggest that many of the initially lagging chromosomes are corrected and then move poleward. The poleward movements were delayed after treatment with Aurora kinase inhibitors. Although they did not formally demonstrate that microtubules were being detached by Aurora B, the simplest model is that Aurora B kinase in anaphase is correcting merotelic attachments to prevent lagging chromosomes from becoming micronuclei.

## Aurora A is localized to spindle poles where it provides spatial information for spindle functions in prometaphase

**FIGURE 5:**
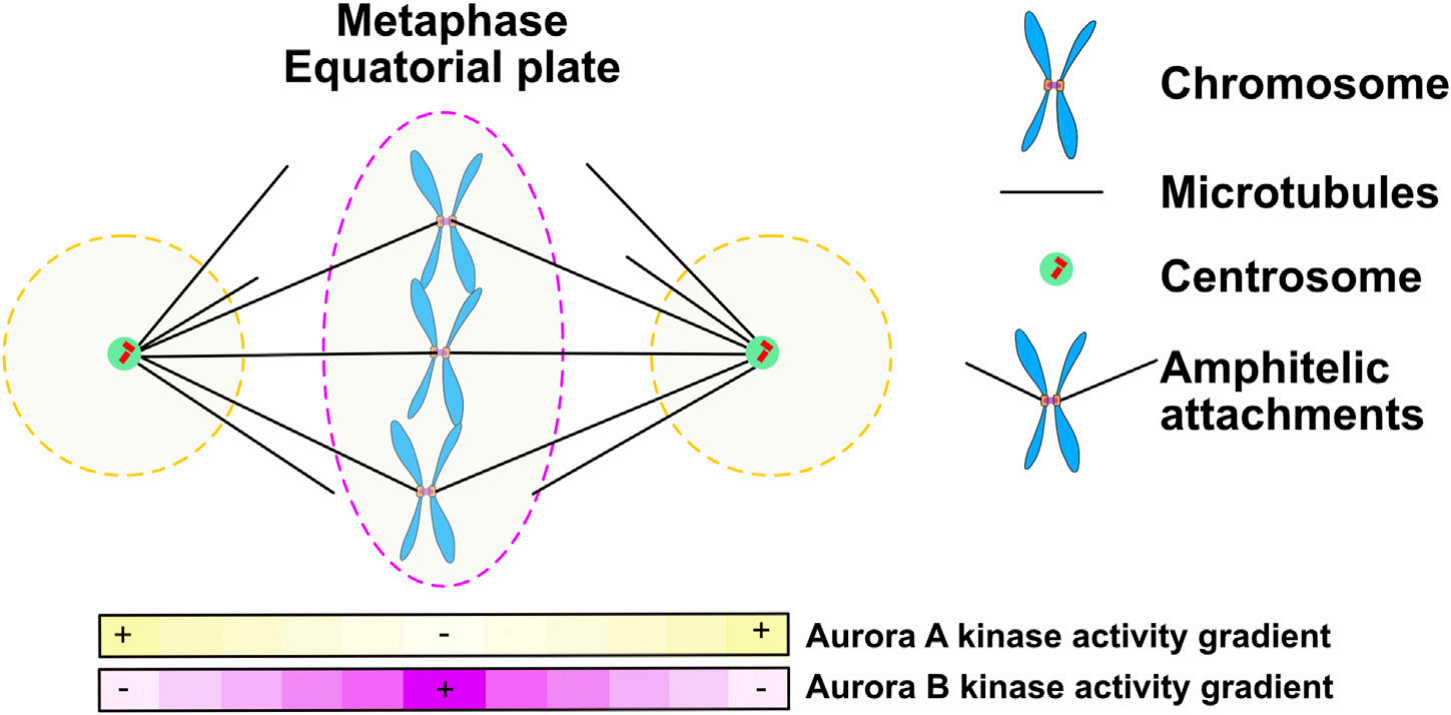
During prometaphase and metaphase Aurora A forms gradients at the poles of the mitotic spindle while Aurora B moves with chromosomes to the metaphase plate and may set up a central gradient. Figure representation: Spindle poles, green circle; Chromosome arms, Blue; Kinetochores, Orange rectangles; Microtubules, Black lines; Aurora A kinase activity gradient within dashed black lines; Ampitelic attachments; two sister kinetochores attached by microtubules from opposite poles; Aurora B kinase activity gradient within dashed red lines; Pink heat map represents Aurora B kinase activity gradient; Yellow heat map represents Aurora A kinase activity gradient.

Aurora A is a kinase that is highly related to Aurora B but the two kinases localize to distinct subcellular regions during mitosis (Figure 5). From prometaphase to anaphase Aurora A localizes to the two poles of the spindle, which are the regions of the mitotic spindle close to centrosomes. Although the Aurora A and Aurora B kinases phosphorylate similar consensus sites *in vitro*, both kinases have accessory subunits that target them to subcellular structures providing substrate specificity. Here we will highlight growing evidence that Aurora A provides spatial information about the location of spindle poles and that Aurora A phosphorylation controls events when substrates come close to the spindle poles. However, we note an important distinction between Aurora A and Aurora B is that soluble gradients of Aurora A kinase activity have not been measured, which suggests that Aurora A only phosphorylates proteins in close proximity to the polar spindle microtubules, which recruit the kinase.

**FIGURE 6:**
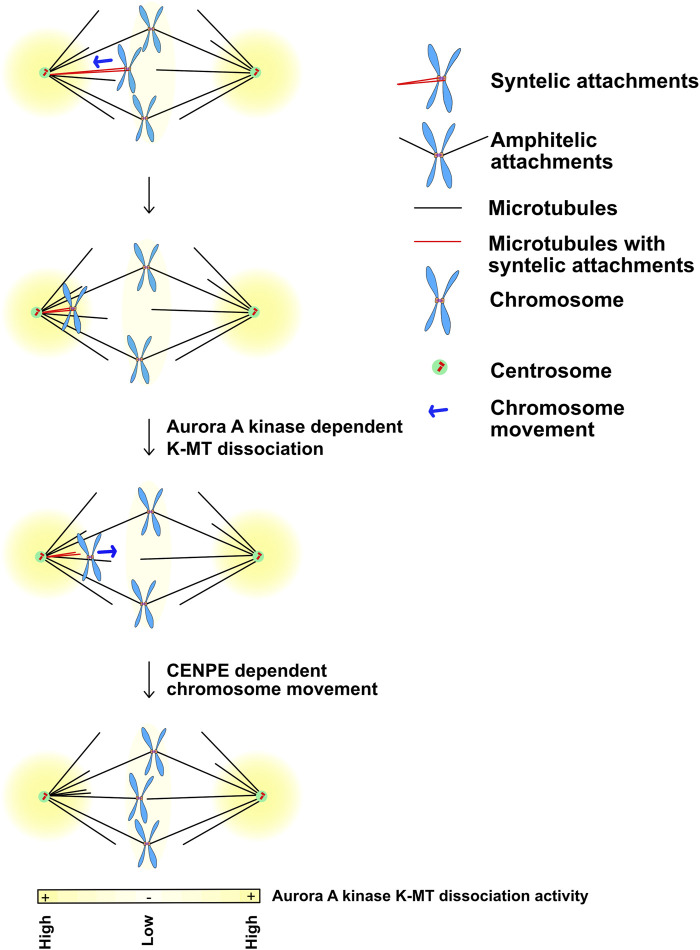
Movement of chromosomes into the Aurora A kinase activity gradients at poles may disassemble Ndc80 dependent kinetochore-microtubule interactions during prometaphase. Yellow: Aurora A kinase localizes and generates kinase activity gradients at the spindle poles. Kinetochores that acquire syntelic MT attachments (in red) are pulled towards the spindle pole while the majority of the chromosomes that obtain bipolar-amphitelic attachments align at the metaphase equatorial plate. High Aurora A kinase activity at the poles phosphorylates outer kinetochore KMN substrates including Ndc80, which allows them to release bound microtubules. It is likely that the chromosomes with lost kinetochore-microtubule interactions translocate back towards the equatorial plate by the plus-end directed kinesin CENP-E until they reach a lower Aurora A activity region that would allow Ndc80 to bind microtubules again. Figure representation: Spindle poles, green circle; Centrioles, red cylinders; Chromosome arms, blue; Kinetochores, orange rectangles; Microtubules, black lines; Syntelic attachments, sister kinetochores attached to microtubules from one pole, red microtubules; Amphitelic attachments, sister kinetochore attached to microtubules from opposite poles; Chromosome movement, blue arrow; Yellow heat map represents Aurora A kinase activity gradient. Abbreviation: K-MT, Kinetochore-Microtubules; MT, Microtubules.

To properly segregate chromosomes every mitotic chromosome must achieve bipolar kinetochore-microtubule attachments meaning that the microtubules bound to one sister kinetochore are directed towards one pole while the kinetochore-microtubule attachments of its sister emanate from the opposite pole. A central function of Aurora kinases during prometaphase and metaphase is to correct improper kinetochore-microtubule attachments and failure to do so is a major source of lagging chromosomes during anaphase. It is generally accepted that Aurora B kinase phosphorylates the Ndc80 protein to release improper kinetochore attachments but there are also observations that suggest that Aurora A can also correct kinetochore -microtubule attachments. Classic experiments showed that syntellically attached chromosomes (both sister kinetochores attached to microtubules linked to the same pole), move to poles, then correct attachments and then realign to the metaphase plate (Figure 6) (Lampson et al., 2004). Work from the Lampson and Maresca laboratories demonstrated that Aurora A at spindle poles can phosphorylate Ndc80 to release these attachments (Chmátal et al., 2015; Ye et al., 2015). Current models suggest that the amount of Aurora kinase phosphorylation on Ndc80 acts as a rheostat to control kinetochore-microtubule attachments. Cells have to limit the amount of Aurora B phosphorylation on kinetochores so that proper attachments can be achieved. Chromosomes near poles require both Aurora A and B to release kinetochores (Barisic et al., 2014) which suggests that the Aurora A at poles may directly phosphorylate Ndc80 and the combined activity of Aurora A and Aurora B drives enough phosphorylation to release chromosomes. Alternatively, Aurora A may hyperactivate Aurora B or control MPS1 which was recently shown to be a central regulator of kinetochore-microtubule release (Hayward et al., 2022).

Most of the events that we have discussed have involved chromosomes but a recent study demonstrates that microtubule motors are also controlled by spatial regulation from Aurora kinases (Janczyk et al., 2023). Accurate chromosome segregation requires that all kinetochores make proper end on attachments so that the sister chromatids can be pulled apart during anaphase. Kinetochores that lack microtubule attachments generate a signal that blocks the onset of anaphase known as the spindle checkpoint (SAC, also known as the spindle assembly checkpoint or mitotic checkpoint). Once kinetochores make the attachments, the signal must be quickly eliminated, which is mediated by the cytoplasmic microtubule motor dynein. During mitosis dynein localizes to unattached kinetochores that generate the spindle checkpoint. Once end-on kinetochore-microtubule attachments are established, the mitotic checkpoint signal is silenced by dynein mediated translocation of the checkpoint proteins down microtubules toward the spindle poles (Howell et al., 2001; Howell et al., 2004). This process is known as the spindle checkpoint stripping.

The dynein microtubule motor carries the kinetochore stripped SAC proteins down the microtubules toward the poles, however, the SAC proteins are released from dynein before they reach the spindle poles (Howell et al., 2001). Notably, this is not because dynein cannot walk all the way to poles as classic work demonstrated that dynein can carry the spindle checkpoint proteins to the poles in the presence of treatments that lower the ATP concentrations in the cell (Howell et al., 2001). The idea is that low ATP concentrations are sufficient for dynein motor activity but there is yet another mysterious ATP dependent step that releases dynein. Recent work from our lab (Janczyk et al., 2023) identified Aurora A kinase as the protein that regulates the release of SAC protein by dynein. Moreover, we realized that spindle checkpoint stripping is an outstanding system to test the hypothesis that Aurora A kinase activity provides a spatial signal from spindle poles. This is because it is well established that the SAC proteins are stripped off the kinetochores and are subsequently carried towards the poles where they are released. Remarkably, the brief inhibition of Aurora A allows SAC proteins to walk all the way to the poles. In addition, we identified the phosphorylation target for this regulation, which is a new Aurora site on the dynein activator Ndel1. Cells expressing non-phosphorylatable mutants of Ndel1 also accumulate a SAC protein at the spindle poles.

Aurora A near poles also regulates dynein to control spindle formation. We obtained a clue from prior literature that the microtubule cross-linking protein NuMA is carried to the poles by dynein and this deposition is regulated by Aurora A (Gallini et al., 2016). However, unlike the SAC proteins, NuMA remains at the poles where it binds microtubules after it is released from dynein (Gallini et al., 2016). Non-phosphorylatable mutants of Ndel1 also accumulate additional NuMA at the poles (Janczyk et al., 2023). Chromosomes can use dynein to move to the pole and must regulate dynein to allow a plus ended directed motor, CENP-E, to subsequently align chromosomes. Aurora kinases have been implicated in this release (Barisic et al., 2014), and it would be of interest to test if Ndel1 phosphorylation also controls dynein in this context. Aurora A inhibition also allows accumulation of the dynein subunit p150 glued at poles in anaphase cells, which suggests Aurora A inhibits dynein through anaphase (Reboutier et al., 2013). Together these studies argue that Aurora A localizes to the spindle poles where it provides spatial signals that are used for the dynein mediated transport of the spindle checkpoint proteins, chromosomes and spindle pole focusing factors.

## Future studies

**FIGURE 7:**
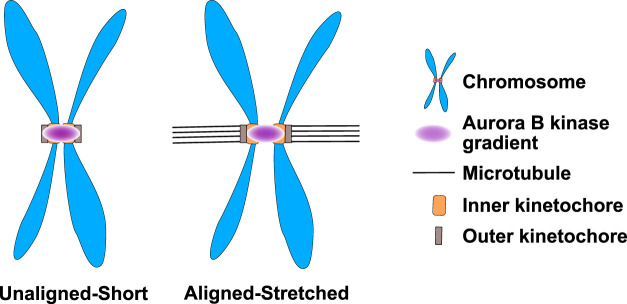
Aurora B kinase may form small kinase gradients at the inner centromere of prometaphase/metaphase chromosomes that differentially phosphorylate kinetochores depending upon their kinetochore-microtubule attachment status. Aurora B kinase in prometaphase and metaphase is localized to the region between kinetochores known as the inner centromere. It has been proposed that Aurora B generates gradients of activity from inner centromeres. The inner centromeric chromatin has spring like activities and is stretched when kinetochores make attachments because the microtubules generate an outward force. Thus, kinetochores in unattached chromosomes would be in a higher Aurora kinase state than the kinetochores that make attachments. Figure representation: Chromosome arms, blue; Inner-kinetochores, orange rectangles; Outer-kinetochore, grey rectangles, Microtubules K-fibers, Black lines; Aurora B kinase activity gradient at the inner centromere, pink.

We have highlighted studies that argue that a key role of Aurora kinases is to localize to either the center of the anaphase spindle or poles of the prometaphase spindle and provide spatial information to proteins about the proximity of these locations. All of these examples suggest roles in defining location(s) along the entire spindle length. Detecting these events is experimentally feasible because of the large size of the mitotic spindle. Aurora B during prometaphase and metaphase localizes to the inner centromere regions between the chromosomes. However, the key substrates of Aurora B are at the kinetochores. It has long been argued that Aurora B might provide spatial information defining the central region of mitotic chromosomes and kinetochores could use these spatial cues to measure the pulling forces of microtubules that displace kinetochores outwards and increase the distance between kinetochores (Figure 7). There are data supporting these models but these data are more controversial, mostly because the short distances (∼1.5 µm) between the kinetochores makes testing spatial control more challenging although there are compelling data that kinetochores respond to distinct levels of kinase activity (Liu et al., 2009). Determining whether and how Aurora B generates spatial information at the center of each mitotic chromosome remains a critical question and an area of future studies.

Another outstanding question for the future research will be to ask if the spatial roles of Aurora kinases are disrupted in cancer and drive chromosomal instability. Aurora A and B kinases and their regulators are the most overexpressed proteins in highly aneuploid breast tumors (Sen et al., 1997; Pfister et al., 2018). Moreover, overexpression of the transcription factors that control the expression of these proteins is sufficient to lower the fidelity of mitosis in non-transformed vertebrate tissues, arguing that these events are sufficient for CIN (Pfister et al., 2018). Interestingly, the dysregulation of the mitotic program and large numbers of aneuploid chromosomes are highly correlated with loss of TP53 function, which is consistent with the important concept that a key function of P53 is to kill or senesce cells that have elongated mitosis or missegregate chromosomes (Stukenberg, 2004; Uetake and Sluder, 2004; Uetake and Sluder, 2010; Tang et al., 2011; Santaguida and Amon, 2015; Lambrus et al., 2016; Santaguida et al., 2017; Pfister et al., 2018). It is unclear if the role of Aurora kinases as spatial information beacons is the key function that is disrupted in tumors to lower the fidelity of mitosis since the kinases have multiple functions. The field needs better tools to separate the roles of Aurora kinases in spatial regulation from the many functions of these fascinating kinases.
